# Music Listening and Homeostatic Regulation: Surviving and Flourishing in a Sonic World

**DOI:** 10.3390/ijerph19010278

**Published:** 2021-12-27

**Authors:** Mark Reybrouck, Piotr Podlipniak, David Welch

**Affiliations:** 1 Faculty of Arts, University of Leuven, 3000 Leuven, Belgium; 2 Department of Art History, Musicology and Theater Studies, IPEM Institute for Psychoacoustics and Electronic Music, 9000 Ghent, Belgium; 3 Institute of Musicology, Adam Mickiewicz University in Poznań, 61-712 Poznan, Poland; podlip@poczta.onet.pl; 4 Institute Audiology Section, School of Population Health, University of Auckland, Auckland 2011, New Zealand; d.welch@auckland.ac.nz

**Keywords:** homeostasis, adaptive behavior, musical-aesthetic experience, musical reward, hedonic pleasure, eudaimonic enjoyment

## Abstract

This paper argues for a biological conception of music listening as an evolutionary achievement that is related to a long history of cognitive and affective-emotional functions, which are grounded in basic homeostatic regulation. Starting from the three levels of description, the acoustic description of sounds, the neurological level of processing, and the psychological correlates of neural stimulation, it conceives of listeners as open systems that are in continuous interaction with the sonic world. By monitoring and altering their current state, they can try to stay within the limits of operating set points in the pursuit of a controlled state of dynamic equilibrium, which is fueled by interoceptive and exteroceptive sources of information. Listening, in this homeostatic view, can be adaptive and goal-directed with the aim of maintaining the internal physiology and directing behavior towards conditions that make it possible to thrive by seeking out stimuli that are valued as beneficial and worthy, or by attempting to avoid those that are annoying and harmful. This calls forth the mechanisms of pleasure and reward, the distinction between pleasure and enjoyment, the twin notions of valence and arousal, the affect-related consequences of music listening, the role of affective regulation and visceral reactions to the sounds, and the distinction between adaptive and maladaptive listening.

## 1. Introduction

Music listening is an evolutionary achievement that has its origins in a long history of cognitive and affective-emotional functions, which are themselves grounded in basic homeostatic regulation [1,2]. A biological conceptualization of auditory processing places musical understanding and interpretation as a way of adapting to the sonic world in the pursuit of internal equilibrium [3,4]. This is the neurobiological and psychobiological approach to musical sense-making as “coping with the sounds”, which, on the one hand, aims to steer clear of those stimuli that are considered harmful for the maintenance of basic homeostatic level setting (see below) and, on the other hand, is also in search of stimuli in the optimal zone of stimulation. The latter may have the potential to render sensory information into percepts that have meaning in the context of listeners’ interactions with the sounds. Coping, in that view, is not only reactive behavior, but involves making sense of the sonic environment as well, ranging from overt physical reactions to mental and cognitive operations. Arguing on these lines, we theorize that coping mechanisms became a part of our musicality, which is also composed of many other adaptive abilities, such as the ability to synchronize our movements or to recognize musical pitch. Therefore, it may contribute to our experience of music [5]. Our (musical) brain, in this view, evolved under evolutionary pressure to read the conditions of our body and manage our internal physiology: to survive, reproduce, and flourish. This requires a controlling mechanism to maintain the physiology and direct behavior towards conditions that make it possible to thrive. Emotions can be seen to mediate between physiology/behavior and surviving/flourishing [6]. Their evolutionary history has elementary homeostatic beginnings and shows a gradual development as brains became more complex, with a gradual expansion of limbic and telencephalic/neocortical structures. There is a rich interplay between bodily changes, affect, and cognition in the experience of emotions. The emotive response to the sonic world in general is also present for music [5,7].

## 2. Setting the Stage: Naturalizing Aesthetics

Recent research on music processing has seen the beginning of a paradigm shift, initiated by the *naturalization of aesthetics*, which studies the aesthetic experience from a biological and adaptive perspective. It is an approach that starts from neuroscience and evolutionary biology and that challenges the distinction between art and non-art [8,9]. Several strands to this kind of investigation can be distinguished in this regard: a strand that studies the feelings of liking, preference, pleasure, and beauty, embedded in Berlyne’s tradition of experimental aesthetics [10,11,12]; a strand based on emotional psychology, for example, revolving around discrete emotional states such as enjoyment, interest, anger, and disgust [13,14]; and a strand that studies unusual states and peak experiences, such as aesthetic chills, feeling touched and moved, losing track of time, and the experience of awe, absorption, and detachment from surroundings [15]. Additionally, there is also the emerging field of *neuroaesthetics*, which tries to identify the neural correlates of aesthetic processing. Starting from the seminal work of Zeki [16] it can be defined as the inquiry into the neurobiological substrates of the aesthetic experience. The principal goal is the empirical study of the underlying brain mechanisms that are involved when objects or events are experienced with an aesthetic attitude [17,18]. It focuses mainly on the perception and production of, and responses to, works of art or objects of aesthetic value. Such an aesthetic concern, however, is not limited to objects of art, but should be generalizable to the communication and experience of spiritual, ethical, and social meaning [19,20].

The neuroaesthetic approach has also been applied to the realm of music [21], with the *musical-aesthetic experience* as an example [22,23,24,25,26]. This has been defined by Brattico and Pearce in operational terms as “[an experience] in which the individual immerses herself in the music, dedicating her attention to perceptual, cognitive, and affective interpretation based on the formal properties of the perceptual experience” [24] (p. 49). Such an experience may thus involve an aesthetic attitude, encompassing aesthetic judgment, aesthetic emotions, and preference along with some degree of intentionality, affective expectations, and dedicated attention, with three major outcomes, namely recognition and induction of emotions, aesthetic judgment, and liking and preference [27].

All these processes typically combine to form a genuine aesthetic situation. It is necessary, moreover, to consider not only the properties of the music, but also those of the listener (such as his/her expertise, internal state, mood, and personality and attitude) and the listening situation (social context and concurrent tasks) as constituting parts of the experience. There is, as such, no strict linear and causal relation between the music as a stimulus and the reactions of the listener. There are, however, some psychophysical and physiological constraints, especially at the lower levels of sensory processing, and some primary reactions to the music, which seem to point in the direction of some form of causal and linear relationship [5,28]. This holds, in particular, for our dispositional toolkit for coping with the sonic world, as exemplified in evolved survival-related behavioral reactions to sudden changes in signal intensity from the environment, which may be associated with potential hazards and opportunities. Typical examples are the orienting response, the acoustic startle response or startle reflex, and to some extent also the myogenic vestibular evoked potentials [29,30,31,32,33]. Most other reactions, however, are learned and acquired and are the outcome of an individual learning history. Yet the link with ancestral adaptive behavior is looming at all stages of processing, even at the higher levels of aesthetic processing.

It has been found, in this regard, that the brain areas that mediate aesthetic responses to objects of art overlap with those that monitor the appraisal of non-art objects of evolutionary importance. Examples are the appeal and desirability of food and the attractiveness of potential mates. Aesthetic processing thus seems to co-opt those neural systems that subserve such adaptive assessments, and, as such, it broadens its scope from a rather limited and unbalanced focus on positive valence phenomena, as advocated in the aesthetics of Enlightenment with a focus on beauty and the sublime, to a broader approach that spans a continuum from negative emotions, such as dislike and disgust, to positive ones, such as awe and ecstasy. Aesthetic processing is reliant on a general cognitive process that is used in other object- or event-related appraisals. The ultimate goal of these appraisals is to adapt our behavioral responses by comparing the estimation of subjective interoceptive states and exteroceptive perception so as to address our basic needs [8].

Assessing the valence of incoming stimuli is only one component of adaptive behavior. The strength of the stimuli and the extent to which they arouse the central nervous system are just as important. The twin notions of *arousal*—operationalized in terms of the activation–deactivation continuum, ranging from sleep to frenetic excitement—and *valence*—ranging from unpleasant to pleasant in the pleasure–displeasure continuum—describe the extent to which stimuli are assessed with respect to both their quality and intensity, with as privileged a domain as the realm of emotions and emotional states. Both arousal and valence have been identified as the principal dimensions of *core affect* [34,35], which has been defined by Lindquist et al. as a “mental representation of the bodily changes that are sometimes (but not always) experienced as feelings of hedonic pleasure and displeasure with some degree of arousal” [36] (p. 25). Such an initial, mainly unconscious, stage of affective processing is realized by our visceral and peripheral sensory systems, relying mainly on subcortical structures that are related to the limbic system. These initial feelings may become conscious emotions, however, after conceptual mediation, which requires the use of language, executive attention, episodic memory, and categorization processes, all of which are controlled by cortical structures [27]. This echoes the concepts of low-road (via the limbic system) and high-road (via the cortex) that LeDoux used to capture emotional processing. The low-road provides a rapid but crude representation and the high-road provides a slower but more elaborate representation [37,38].

Arousal and valence, as the principal dimensions of core affect, are also central components of the aesthetic experience, which makes it possible to ground the latter more generally in standard theories of emotions. This is an important step towards naturalizing aesthetics with, as a central topic of research, the distinction between “basic emotions” and “aesthetic emotions” and the related difference between “utilitarian” and “aesthetic emotions”. Basic or genuine emotions are supposed to reflect environmental states, as well as cognitive appraisals and the accompanying physiological processes that are triggered by them. They may act as powerful motivators for behavior and are adaptive mechanisms, by definition [39]. Aesthetic emotions include emotions such as wonder, transcendence, nostalgia, tension, and awe, which typically do not trigger immediate goal-directed actions [40,41].

Musical emotions combine aesthetic and non-aesthetic emotions [7,42,43]. They have been described in the Geneva Emotional Scale (GEMS), embracing nine distinct categories, such as wonder, transcendence, tenderness, nostalgia, peacefulness, power, joy, tension, and sadness [41]. Music may thus function as a reward system that capitalizes on emotional reactions and aesthetic responses by eliciting the limbic and paralimbic activations that are involved in affective processing, with the distinction between rewarding or aversive properties of the stimuli as a major dividing line [44]. It can be questioned, however, to what extent aesthetic emotions are grounded in survival-related adaptive behavior. There is currently, a whole strand of research that defines them as exploratory behavior in a changeable environmental world. Therefore, they entail a unique manner of engagement with the sonic world so as to produce adaptive and flexible behavior in terms of coping with the sounds [5,45].

## 3. Neural Underpinnings of Coping with the Sounds

Music is a vibrational phenomenon that impinges upon our body and our brain. There are three possible levels of description: the acoustic description of the sounds; the neurological level of processing; and the psychological correlates of this neural stimulation. These levels are not separate, but are intertwined, with the neural level functioning as a vital connecting link for receiving, analyzing, and evaluating the incoming information. Together with the sensory system for monitoring the acoustic environment, the auditory neural system provides our innate dispositional machinery for coping with the sounds [5] and makes it possible to conceive of music listening in terms of adaptive behavior. This means that listeners evolved coping responses to sounds available prior to music so they listen to music in part because of these, but also to exercise these mechanisms. Listening, in that sense, both draws on existing adaptations and requires adaptive listening. Coping with sounds, then, is only one of the adaptive functions besides the facilitation of social bonding [46,47], enhancing sexual selection [48,49], credible signaling [50,51,52], and the facilitation of mother–infant bonding [53].

### 3.1. The Concept of Coping and Its Mechanisms

The concept of *coping* is of primary importance here. In a general sense, it is a survival mechanism for living organisms through their interaction with the environment. Therefore, it may contribute to the maintenance of a state of equilibrium with regard to both the internal and external environment. To achieve this state of equilibrium, our body can rely on a whole complex of organ systems—such as the sensory system, the musculoskeletal system, the nervous system, the cardiovascular system, the excretory system, the respiratory system, the digestive system, the endocrine system, the integumentary system, and others—whose combined actions aim to regulate our “internal milieu”, to adopt Bernard’s term [54], and which work together as a self-regulating process to maintain internal stability while adjusting to changing external conditions.

Our body, however, is also an open system that is in continuous interaction with its environment. It is important, therefore, to monitor and alter its state to stay within the narrow limits of the body’s *operating set-points*. These are the desirable reference or baseline physiological quantities that refer to the optimum values for blood pressure, pulse rate, breathing frequency, body temperature, blood sugar, pH, oxygen and carbon dioxide level, fluid balance, etc. This means that the internal metabolic processes and the outward-directed activities in response to sensory inputs must be balanced and fine-tuned against optimum target functioning. Such pursuit of a controlled state, with a dynamically equilibrated and balanced internal milieu together with its underlying physiological processes, has commonly been known as *homeostasis* since Cannon first used this term [55] (see also [56,57]).

The maintenance of this balance is fueled by two sources of information: one which originates from within the organism through interoceptive pathways; and another which derives from the external environment through exteroception. Translated into the domain of music, this should mean that listeners attune themselves not only to the music as an external stimulus but also to the physiological reactions of their body. The latter are not always overt and manifest, but it is possible to learn to read the body and respond to its minor changes.

Listening, in this homeostatic view, can be highly adaptive and goal-directed by searching out stimuli that are valued as beneficial and worthy, or by trying to avoid those that are considered annoying and harmful. It is related to coping behavior in general by choosing between two alternative directions: promoting those behaviors and searching for stimuli that are conducive to optimal or better functioning; or avoiding those that impede such functioning. There is some relation with the motivational systems of *approach* or *avoidance* [58], which brings with it the role of musical pleasure and reward and the complex coupling with the neurochemistry of emotions [59]. Central in this approach is the role of the autonomic nervous system and its mediating influence on the secretion of the glands. It means that music can trigger the hormone system with the release of substances that either increase or decrease the above-mentioned operating set-points. Music, then, can be a source of pleasure and enjoyment, but it can be experienced as a possible stressor as well. A distinction should be made, however, between stress that is triggered by harmful stimuli or activities and stress that is a kind of challenge, which can even be experienced as a positive adaptive reaction to beneficial stressors. Selye has introduced the notions of *distress* and *eustress* in this regard [60,61,62], with the latter representing the pleasant feeling of fulfillment while simultaneously avoiding the harmful consequences of damaging stress. It is a condition that values efforts in terms of positive valence with effects that guarantee no damaging outcomes and stressors that do not exceed the capacity for maintaining or restoring the process of homeostasis [5].

The literature on coping behavior, however, has focused mainly on avoidance behavior. There is a whole dispositional machinery to cope with potentially threatening stimuli, such as the orienting response, the startle reflex, and the fight and flight reaction, all of which are warning or alerting reflexes that make it possible to avoid harmful or annoying stimuli as much as possible. Further, such avoidance behavior is typically oriented to acute stressful situations. Yet there are major long-lasting effects which may be damaging in insidious ways, with cases of hearing loss or hearing damage as typical examples together with symptoms that have been grouped under the umbrella term of vibroacoustic disease (VAD) [63]. Adaptive coping, then, should focus not merely on acute shifts in reactive activity in response to specific stimuli, but should try also to avoid the chronic and cumulative elevations in physiological activity outside of the basal operating ranges [64]. This entails a shift from avoidance behavior to the celebration of optimal functioning that broadens a narrow biological conception of hearing as merely an acoustic warning system with a primary function to recognize the energy fluctuations in the environment [39]. It is a conception that goes beyond basic homeostatic functioning by promoting optimal navigation in the environment in search for opportunities in the world, thus allowing a positive redefinition of coping behavior in terms of the search for “pleasure” and “enjoyment”. Both terms, however, are not identical in the sense that pleasure refers to the kind of good feeling that comes from the satisfaction of homeostatic needs such as hunger, sex, and bodily comfort, whereas enjoyment refers to those feelings that go beyond the limits of homeostasis, such as an artistic performance, a stimulating conversation, or an athletic event [65] (p. 12). It is thus possible to go beyond the management of physiological responses to generalize from fundamental sensory pleasure in an attempt to understand the larger hedonic brain principles that contribute to happiness. What matters here is the relation of *hedonic components*, such as pleasure or positive affect, to *eudaimonic components* [25,66], with a shift from mere reactive responses to auditory stimuli to the appreciation of a full-fledged aesthetic experience [26,67].

The hedonic experience focuses rather narrowly on the experience of pleasant feelings with a healthy balance between positive and negative affect. It takes the view, advocated by Bentham, that pleasure is the only thing to be pursued as opposed to pain, which is the only thing to be avoided [68]. The eudaimonic experience, on the contrary, aims toward broader goals, such as the realization of our potentials and personal growth. It is inspired by Aristotle’s instigation to realize our own daimon or true nature. Both experiences can be conceptually distinguished, but in practice they can go hand in hand. Therefore, there are activities that give rise to both eudaimonic and hedonic enjoyment, while some others are hedonically enjoyed without giving rise to eudaimonia and others giving rise to eudaimonia without being hedonically enjoyable [69,70].

### 3.2. Musical Pleasure and the Reward System

The transition from hedonic pleasure to eudaimonic enjoyment does not happen without a struggle. It depends on the tension between the two types of neural mechanisms, manifested in the *sensory hypothesis* and the *conceptual hypothesis* of musical pleasure [27]. The sensory mechanisms determine the succession of neural events from the periphery to the central nervous system. They are the primary windows to the world, which provide characteristic and specific ways of processing information, allowing the experience of the qualitative and quantitative—the prothetic (how much)–metathetic (which kind) distinction [71] aspects of the world. There are, however, levels of qualitative experience with a graded nature of conscious experience, ranging from unconscious or preconscious to totally conscious and deliberate processing, and with a further distinction between conscious sensation, conscious perception, and even higher forms of cognition [72]. In neurological terms, there are cognitive mechanisms that originate from the prefrontal and association cortices in the brain and that modulate these lower-level peripheral reactions. There is, therefore, a possible transformation of sensory pleasure into conscious hedonic feeling, such as the liking of a musical piece through the mediation of the higher-order structures of the brain.

A distinction is to be made, in this regard, between the two ways in which “higher” and “lower” levels of processing may interact. Firstly, the perception of sound is not a one-way street. Top-down interpretations interact with the bottom-up flow of information from the periphery to the brain, and this top-down influence occurs throughout the auditory system so our perceptions are never purely sensory. The extent to which the top-down “expectations” are met or violated by the bottom-up “evidence” has been suggested as being one of the sources of variance in the enjoyment of music [73]. The second type of distinction between high and low occurs in the parallel, yet qualitatively different, processing carried out in the low-road (limbic system) and high-road (cortical) response to music [37]. There are multiple anatomical connections of the auditory pathways to the central nervous system. Alongside the “high-road” pathways from the inner ear to the auditory cortex, there are the “low-road” pathways to the reticular activating system, other parts of the limbic system and brain, the autonomic nervous system, and the neuro-endocrine system, which together control the physiological, emotional, and behavioral responses of the body [74]. In both cases, the two forms of high/low processing ultimately come together as an interaction, which itself is the combined response to the music. Furthermore, both sets of processing must happen simultaneously, in that both the low-road and the high-road have bottom-up input and apply top-down perceptual systems.

**Figure 1:**
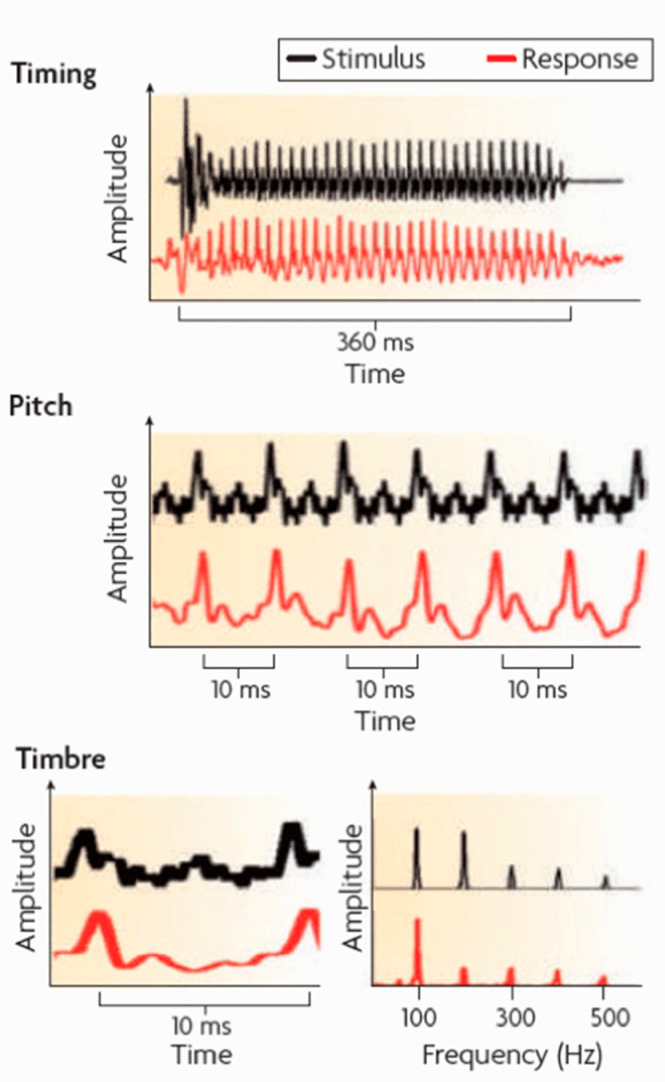
Auditory evoked neural response to temporal characteristics, frequency, and spectral features as the representations of timing, pitch, and timbre in the human auditory brainstem, depicted as changes in amplitude across time (**top, middle** and **bottom left**) and as spectral amplitude across frequency (**bottom right**). Adapted with permission from Ref. [75]. (Copyright 2010 Nature Publishing Group, Macmillan, Berlin, Germany; License number: 5213020142320).

The role of the brainstem has been somewhat underrated in this regard. It is crucial for the fast interpretation of sounds in terms of their location, level or informative value [75,76,77]. The brainstem preprocesses and encodes the basic information-bearing elements in music, such as pitch, timing, and timbre, before there is any perception or cognitive engagement. Therefore, it reflects the current state of the nervous systems in response to acoustic stimulation as a kind of neural coding of selected sound. It promotes signals that can be interpreted as pitch, timbre, loudness, and rhythm in music (Figure 1).

The brainstem is one of the evolutionarily oldest systems of the human brain. One of its many functions is to promote arousal and excitement through connections between the auditory pathways and nuclei to the *reticular formation* or *reticular activating system*. As a whole, it has a major role in the modulation of the experience of sound, the coupling with other sensory systems, the initiation and control of motor activity, autonomic arousal, sleep and wakefulness, and emotions [78]. It monitors all ascending pathways in the brainstem and projects impulses to various parts of the brain in case of extraordinary sensory input—such as, e.g., a loud sound—to trigger a state of alertness [79]. Therefore, it partially explains why people enjoy loud music [73,80,81]. Its function, however, cannot be seen in isolation from the human brain as a whole, which is an evolutionary accretion of three layers that have evolved phylogenetically, namely the reptilian brain, the limbic system or primitive/paleomammalian brain, and the neo mammalian brain or human cortex, and that have been termed the “Triune” Brain [82,83].

Acoustic stimuli are not merely vibratory energy. They can also be the source of musical pleasure—this is the sensory hypothesis of musical pleasure—with several neural mechanisms that may build up to generate enjoyment. An aesthetic experience, in this sense, adds layers of sense-making and consists of many interconnected levels of processing, such as a potential association between aesthetic awe and arousal, and an integration of perceptual, evaluative, and reward components [84]. Research to uncover the neural underpinnings and the physiological bases of aesthetically moving experiences has found that activity in the reward circuit plays a major role in this experience [18]. Several brain regions have been identified in this regard, such as the anterior medial prefrontal cortex, the caudate/striatum, and the reticular formation. Since the striatum appears to reflect reward valence and the reticular formation reflects arousal, a parallel has been drawn between these and the principal axes of core affect [84].

The overall picture that emerges is quite challenging. It highlights the evolutionary history of musical emotions and the role of the affect-related consequences of music perception, which point to a large number of cortical and subcortical regions in the brain, commonly designated as the *limbic* and *paralimbic belt* [23,85,86]. They include the nucleus accumbens, the ventral tegmental area, and the hypothalamus and insula, which together regulate the autonomic and physiological responses to rewarding and emotional stimuli [87,88]. Most studies, however, have concentrated rather narrowly on cerebral sites and evolutionarily younger telencephalic sites, such as the dorsal and ventral striatum and the amygdala. What has been neglected to some extent, are those brainstem structures that house some auditory processors as well as some core mechanisms of homeostatic regulation, such as physiological arousal [89].

Physiological arousal is mediated by the activation of the sympathetic nervous system and relies on dopaminergic synapses in hypothalamic pathways. Music can influence brain function through the modulation of *dopaminergic activity* within the reward circuit, and this links dopamine release with intense musical pleasure [24]. Even if there is not yet conclusive evidence about the role of the intrinsic qualities of music in this regard [90], there are some music-specific features, such as pitch centricity [91], that are related to specific responses of the reward system, as in the case of out-of-key notes in a tonal melody [92]. This means that music can function as a mediator, which, via the autonomic nervous system, can influence physiological responses such as heart rate, blood pressure, respiratory rate, body temperature, skin conductance, and muscle tension. This is driven partly via the noradrenergic neurons that regulate cholinergic and dopaminergic neurotransmission [25,59]. Such physiological responses influence the listener’s mood and motivation by strengthening the dopaminergic activity within the reward circuits, and this may further reward and motivate listeners [93].

## 4. Emotional-Affective Level Setting and the Processing of Information

Emotions have been considered as having an evolutionary adaptive function. They can occur outside of awareness, but they are not always devoid of self-direction and deliberate control. This is reflected in two schools of thought [69]: one holds that emotions return to baseline levels after an emotive experience due to homeostatic mechanisms, as thermostats adjust room temperature; the other holds that people possess the self-reflective and agentic capabilities to restore emotional equilibrium. The latter view may also extend to growth or improvement in eudaimonia as the result of coping with adversity [94,95,96].

In what follows, we will argue for a position that includes both processes. In the brain, a core circuit involving the anterior insula and the orbitofrontal cortex appears to process aesthetic perceptions. It involves an interaction between interoception and exteroception, including visceral perceptions [8,97,98]. It is active during the appraisal of valence and may combine the subjective awareness of a current homeostatic state with the perception of objects or events in the environment. Such assignments of valence can be termed as triggered *homeostatic emotions* [99].

### 4.1. Affective Regulation and Visceral Reactions to the Sounds

Music listening can be described in terms of affective regulation. Listening may even have an adaptive and evolutionary meaning in the sense that the brain has evolved to learn to read the conditions of the body and respond accordingly in different situations via the machinery of emotions [6]. Stated differently, emotions are triggered by specific events and have their basis in physiological states so as to increase the ability to respond appropriately to possible threats and dangers in the environment [100].

This can be easily translated into the field of music. Sounds function as a means of contact with this environment and the hearing system has evolved in part as a warning system against possible dangers, in part to assist in acquiring environmental benefits, and in part as a mode of communication. The central nervous system processes the information in the sounds, compares them with previous experiences, and extracts meaning on which appropriate reactions need to be initiated. Environmental sounds may be experienced as normal and acceptable or may possibly alter the homeostatic level setting [74].

This may seem common to many other kinds of conditioned stimuli, but the uniqueness of music lies in the fact that music became part of a more elaborate semiotic tool that is more complex than expressive dynamics. The process of conditioning and the psychological, social, and cultural elements that condition listeners to specific kinds of music provide a kind of color to the experience that makes it distinct from other kinds of conditioning. Even though the process of conditioning may be general, its application in the context of music is much broader and draws upon all the important and influential aspects of our makeup, such as emotions, intellect, sociocultural stance, and spiritual awareness.

Among the reactions in general, those that elicit negative emotions have attracted by far the most attention. Several explanations have been put forward in this regard. Negative emotions appear to be more closely tied to reality than positive ones—this is the known phenomenon of *depressive realism*, which states that sadness encourages more detail-oriented thinking, less judgment biases, and the more realist assessment of the likelihood of certain outcomes [101,102,103]—and they are more urgent in the sense that they reflect immediate problems or objective dangers. Therefore, they must be powerful enough to stop ongoing activities, increase our vigilance, reflect on the behavior and change actions if necessary. Positive experiences, on the contrary, are less obvious in terms of their adaptiveness and may often seem to pass effortlessly [65]. It can be questioned, however, whether this can be generalized to all negative emotions and, in particular, to the negative aesthetic emotions. There is, in fact, a distinction to be made between emotions such as anger and disgust, and emotions such as nostalgia and sadness, which differ considerably with regard to their arousal-inducing potential. Moreover, the whole discussion about the distinction between real-life, simple, utilitarian emotions and so-called aesthetic emotions is still open [15,41,104]. Much is to be expected from additional research on the aesthetic emotions of “awe”, “being moved”, and “wonder” and their alignment with Konečni’s aesthetic trinity of awe, being moved, and thrills [105], and their relation to the evocation of arousal and fine-grained physiological reactions, which are the subject of current research (see [106]).

This brings us to the above-mentioned distinction between *valence* and *arousal* as the primary dimensions of core affect. Our responsiveness level (arousal) can be high or low and the way we value the music can be positive or negative (valence), which makes it possible to position affect in a two-dimensional space [107]. Such a space allows for a multiplicity of possible emotion states. Some of these may be more salient and can be called *peak experiences*, on the condition that there is at least a cognitive element that transforms the person so as to experience intense psychological states that are characterized by the feelings of highest happiness and fulfilment [108]. Mere emotional bursts do not have this transformative power. Peak experiences, moreover, entail two types of reactions, namely one of excitement and high tension, and another of relaxation and stillness (see also [109,110,111].

Arousal can be elicited by the startle response and fight and flight reactions. Startle responses are simple defensive responses to sudden acoustic, tactile or visual stimuli that signal proximal threat. They have been described in detail [112,113,114] and can be potentiated or attenuated by a variety of factors, particularly fear and stress [115,116]. The startle-induced *hypothalamic–pituitary–adrenal (HPA) axis* response to these kinds of stimuli is of particular importance, with empirical evidence for the elevation of the adrenocorticotropic hormone (ACTH) and cortisol concentrations above baseline levels, which are responsible for a heightening of the arousal level in general [117]. Some sounds are more startling than others. Rough temporal modulations of sounds, such as screaming, scratching or breaking glass, may selectively activate the amygdala, which mediates between the threatening stimuli and defense reactions [118,119,120]. Some kinds of music that contain similar sounds can also trigger these reactions [100].

**Figure 2:**
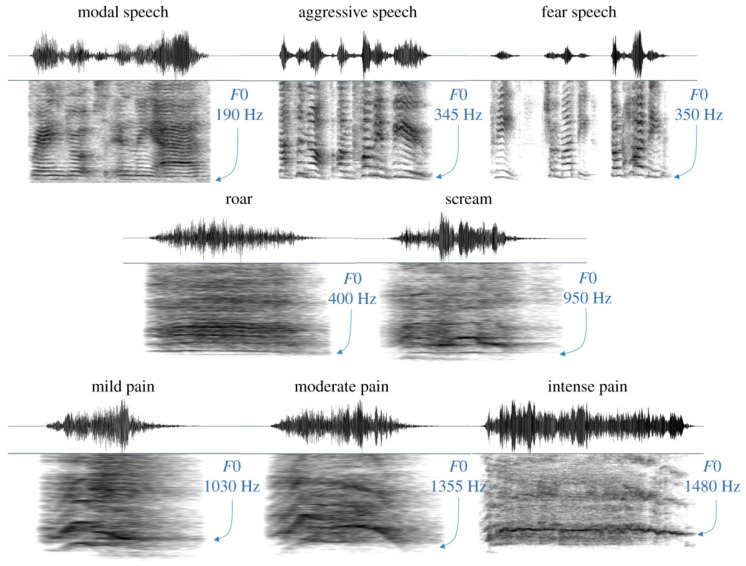
Waveforms and spectrograms representing each of eight vocal types produced by a single representative individual (female, aged 22), demonstrating the high degree of intra-individual variability in F0 across vocal types. Reprinted from Ref. [123].

Human screams, in particular, are one of the most relevant communication signals. They result from the bifurcation of regular phonation to a more chaotic regime, which is a major characteristic of nonlinear dynamics. Bifurcation refers to the transitions between qualitatively different vibratory regimes with a splitting of the periodic behavior of a system, such as the onset of phonation. It means that gradual changes in some control parameter—such as vocal fold tension or subglottal pressure—can lead to abrupt and qualitative changes in the vibratory/acoustic behavior. Chaos, or deterministic chaos, on the other hand, is to be understood as the generation of nonperiodic, irregular vibrations, which are characterized by a broadband spectrum with energy at many different frequencies, and which are perceived as being harsh and noisy. Screams, therefore, occupy the roughness region of the modulation power spectrum, which confers to them their alarming nature [121,122]. They are also characterized by a modification of the fundamental frequency (F0), which may increase considerably compared to speech in the same individual. Figure 2 provides an example of a female, aged 22, demonstrating the high variability of intra-individual variability in F0 across distinct vocal types, such as neutral speech, valence speech and nonverbal vocalizations such as screams, roars, and pain cries [123].

This is also the case with some kinds of music—garage rock, punk rock, and heavy metal are typical examples—with overpowering beats, extreme amplification of guitar sounds and other instruments, and a screamed style of singing, where distortion effects mimic the nonlinear characteristics that are typically seen in aroused animal signals and human voices and whose angering effects seem to have a physiologically arousing effect on listeners [124]. Besides the impact of scream-like higher pitches, the celebration of bass culture should also be mentioned in this regard. By focusing on the vibrational transduction of affect and the low frequency drive for sonic dominance, it may function as a trigger for an adrenalin rush. This has been described by Goodman in terms of sonic warfare, with vibrational materialism tended to sonic extremes and an aesthetics of “jouissance of sonic physicality” at the limits of the audible, both at the level of infra- (<20 Hz) and ultrasound (>20 kHz) frequencies [125] (see also [126] for a critical discussion). The depths of low bass frequencies particularly have a kind of “haecceity” or “thisness”, a force of attack and sharpness of edge in comparison to the more tamed, domesticated, and culturally recuperative mid-frequencies of common music [127] (p. 38). In this regard, there have even been attempts to use music as an acoustic weapon and as a kind of no-touch torture by the defense departments of some countries in the past, based on the insight that sound may have an immersive weight, liminal force, and substantive presence that is impossible to escape or deny. The audible then becomes tangible and haptic [126,128].

A common factor of attention-capturing and alarm-encoding sounds is their capacity to ensure communication efficacy. This is realized by acoustically segregating the signals from other communication signals by occupying a privileged niche or restricted portion of the acoustic communication space that corresponds to the perceptual attribute of roughness. Such acoustic roughness is present in natural and artificial alarm signals and facilitates their detection by engaging subcortical structures that are critical to the rapid appraisal of danger [122]. It is realized through their spectro-temporal specificity, as evidenced from waveform, spectrogram, and modulation power spectrum (MPS) representations (see [122] for a technical description). Most notable in this regard are the nonlinear characteristics and roughness of highly aroused animal signals (shrieks, alarm calls, and contact calls) and human voices (screams), as well as the search for “hot” sounds in much contemporary music, which are characterized by the greater density of their spectral contents and distortion effects (more rectangular waveforms) that are responsible for an overblown amplification effect. Such sounds reach their maximal amplitudes and correspond perceptually to harsh, noisy sounds that penetrate noisy environments, thus favoring maximal discriminability and requiring a quick response or attention [121] (see also [129] for a broader biomusicological underpinning of the survival value as a ultimate function). Listeners, accordingly, may display psychophysiological responses to these nonlinearities with measurable autonomic reactions, even when they are not deliberately aware of them [130] (see also [5]).

### 4.2. Emotional-Affective Processing and the Gathering of Information

Affective visceral reactions to sounds may have survival value in the case of threatening environments. They provide a way of coping with the sonic world. Coping behavior, however, has been approached mainly from a negative side. Yet it is also possible to conceive of it as a way of sense-making, relying largely on the mechanism of *exploratory behavior* [5]. There are, after all, three systems to monitor and respond to the environment by focusing our mental processes on incoming stimuli: alerting; orienting; and executive control [131].

The evolutionary machinery of emotions is quite important here, in the sense that the sonic environment—and thus, also music—can be approached in terms of potential *emotionally competent stimuli,* which can be described as objects or situations that are either real or conjured up in our mind and that may act as keys to activate certain parts of our brain that were designed by evolution. They have the power to induce emotions and the chain of physiological events that can bring about changes in the body and the mind, which may ultimately lead to certain feelings [132]. Further, it has been shown that there is a strong interconnection between cognition and emotion as two interrelated aspects of human functioning. Emotions embrace the cognitive as well as sensory processes and they affect aspects of cognition, such as learning, attention, memory, decision making, motivation, and social functioning. It is a notion that is grounded in a neurobiological view of cognition as functioning in the service of life-regulating goals, which is implemented by emotional machinery [6]. Emotional strategies and investment are thus seen as basic forms of decision-making. They are adaptive in the sense that they are triggered by specific events, and their accompanying physiological changes increase the ability to respond appropriately to threats or danger in the environment by means of a balanced interplay between valence and arousal [100].

The role of arousal cannot be overestimated in this regard. It can be defined as a state of the brain or body that reflects its responsiveness to sensory stimulation and is correlated with changes in behavioral, hormonal, and/or neurological activity [107,133], albeit in a nonlinear way. There is, in fact, the danger of overstimulation and the corresponding management of arousal, which has been investigated in the context of psychobiological contributions to experimental aesthetics that define aesthetic appreciation as a function of perceived arousal. Optimal arousal is found with stimuli that occupy the middle ground between novelty and banality [10,134], as suggested by the inverted U-shape curve between arousal and general responsiveness—the Yerkes–Dodson law—that states that the optimum motivation for a learning task decreases with increasing difficulty [135].

As such, it is somewhat related to the feeling of *enhanced attentiveness* as a defining characteristic of heightened affective experiences [109,111]. Though still somewhat elusive, it is challenging to try to relate the regulation of attention with exploratory behavior, arousal, and the seeking of reward [136]. There is, at a neurobiological level, a mesocorticolimbic pathway that is part of a larger general, purpose foraging system in animals, which enables the establishment of adaptive expectations about the availability of reward within the environment [137]. This seeking disposition toward the environment is itself a strong elicitor of dopamine-related hedonic pleasure, even independent of the achievement of reward [138]. Animal (mainly rats and monkeys) and human studies have revealed that the dopaminergic neurons in the ventral tegmental area (VTA) promote arousal either through tonic activity to maintain a baseline level of dopamine release, or through phasic bursts that fire in response to certain eliciting cues, both of which antagonize each other [139,140]. Examples of potential causes of altered dopaminergic firing are unpredicted rewards, prediction errors, novel stimuli, physically salient stimuli, motivational and affective salience, and attention shifts related to approach behavior [137,141,142,143].

Aesthetic emotions have a privileged position in this regard. Their awareness and recognition can be seen as tools for understanding the ever-changing world by adding a conceptual dimension to mere bodily instincts, in the sense that cognitive consonance or dissonance between our knowledge and the world can generate the aesthetic emotions of either satisfaction or dissatisfaction, respectively [27]. Such “cognitive mastering” might alter the affective state with positive effects. It is a crucial stage in information processing, which leads to the aesthetic outcomes of judgments and emotions [12].

Further, cognitive mastering has been shown to activate additional limbic subcortical areas. It explains, to some extent, the positive affect derived from understanding [27] and the role of the affective component of music appreciation and enjoyment by enabling the integration of the brain regions that process natural rewards with those that are involved in high cognition. This interaction allows top-down processes to mold the perception and interpretation of musical stimuli through previous experience and knowledge [18,136,144]. It is a challenging new field of inquiry, with a central focus on the role of dopamine as a crucial neurotransmitter in the reward system in different regions of the brain. The relation between the dopamine release, in cases of listening to music that listeners did or did not deeply enjoy, and their arousal peaks especially has revealed an unexpected functional dissociation between two structures of the basal forebrain; the caudate nucleus is more active during the anticipation of peak emotional experiences, while the actual experience itself is associated with dopaminergic activity in the nucleus accumbens. It thus seems that the emotional experience of music is mediated by distinct anatomical pathways, which play a complementary role [145].

## 5. Music and Health Regulation: Adaptive and Maladaptive Listening

Music listening can contribute to greater well-being but it can also be a factor of stress. Much depends on the nature of the eliciting stimulus as well as on the way of listening. There is a lot of freedom here, but it is possible to take an evolutionary and neurocognitive stance in this regard [146,147], revolving around the hypothesis that the origins of music can be related to the facilitation of social bonding [47], which leads to the relief of anxiety and tension and the feelings of connection to a group [20]. Supportive evidence has shown that music has the power to diminish systemic stress hormone levels [148]. Yet, the enjoyment of music cannot be explained exclusively from the negative side. A more fruitful proximal explanation is an approach that argues for a mechanism of homeostatic regulation with a subtle balance between the avoidance of harmful stimuli and the search for positive and reward-inducing stimuli. Music listening, then, should be defined in operational terms as the search for an optimal allostatic load.

### 5.1. Music as Stressor: The Concept of Allostatic Load

The concept of *allostasis* goes back to Sterling and Eyer [149]. It literally means “stability through change”, and emphasizes the dynamic nature of our internal physiology through continuous and ongoing adjustments and alterations to respond and adapt to the environmental demands. It should be understood within the general findings that all living organisms can respond to stress with a basic reactive pattern that is always the same, which was described by Selye as the “general adaptation syndrome” [150]. It can be considered as a single unified biological system that revolves around Bernard’s concept of maintaining a constant “internal environment” and Cannon’s concept of “homeostasis” [54,55], which tries to keep the basic homeostatic level setting within safe limits by meeting life-endangering situations with adaptive responses. The ability of living organisms to adapt themselves to changes in their surroundings, however, is limited, which means that both adaptability and resistance are relative terms. It makes sense, therefore, to attune ourselves to sound environments and music that provide stimulation in the optimal zone of arousal, allowing us to cultivate an ensemble of positive adaptive reactions to beneficial stressors as well as avoiding possible distress triggered by harmful stimuli or activities.

Allostasis, in this view, can be conceived as the strain on our organs and tissues as the result of repeated fluctuations in physiological responses to perceived threat and multiple forms of adversity, which, in the long-term, can lead to organ breakdown, reduced immune response, elevated cortisol and insulin secretion, and finally disease [151]. Further, there are two possible outcomes of the accumulation of allostatic load: the wear and tear of acute shifts in physiological reactivity in response to specific stimuli (see above); and the chronic and cumulative elevations of level settings outside of the basal operating ranges, which operate mainly in the absence of threatening stimuli and can be assessed by a number of indicators of possible physiological system impairment (see [64] for an overview). This allostatic load, however, is not always harmful. It is possible, in this regard, to conceive of an “optimal allostasis” as the successful adjustment and adaptation to the changing conditions of the environment. This includes not only the maintenance of allostatic load indicators in normal operating ranges, but also the management of specific brain opioids—such as β-endorphins, leucine, and methionine enkephalins—that are helpful in counteracting negative emotions and promoting positive ones.

It can be questioned, in this regard, whether music could be considered as a possible harmful stressor that activates the hypothalamic–pituitary–adrenal (HPA) axis or as an emotional competent trigger that activates those neuroendocrine responses that are related with the reward center and the hedonic hotspots of the brain. In the latter case, it could function as a beneficial stressor, on the condition that it does not exceed the capacity for maintaining or restoring our homeostatic level setting.

There is, in this regard, a distinction to be made between negative/aversive (distress) and positive/rewarding stress (eustress). Even if both are associated with the increased activation of the HPA axis, the effects can be either detrimental, in the case of chronic negative stress, or positive, in the case of coping with a challenging environment [152]. Adaptive listening, in a physiological sense, should try to align itself with an optimal allostatic load so as to operate within an optimal zone of stimulation. This means that listeners should try to avoid physiological stress in terms of an altered homeostasis of the central nervous system functions [74]. This holds in particular for noisy environments and loud music with the lasting effects of hearing loss and histopathological changes, both as the primary neural degeneration of cochlear nerve afferents (spiral ganglion cells) and loss of acoustic hair cells and as subjective annoyance [153,154,155,156]. To the extent that this kind of listening compromises the basic homeostatic level setting, it can be considered as a “maladaptive response style” [157,158].

Living organisms monitor some aspects of their own internal bodily states. Failing to do so can compromise the homeostatic level setting that is necessary for life and smooth functioning. It is thus important to diagnose these changes and develop ways of modulating the internal physiology so as to preserve or re-establish the necessary baseline settings [159] (p. 57). Care should be taken, however, not to reduce maladaptive listening to mere physiological effects. Stress, both positive and negative, has been found to have connections with life experience, emotions, and health outcomes [64]. There may even be sought-after strategies for coping with so-called harmful or threatening stimuli, either through positive adaptive or negative maladaptive reactions [5] with psychological variables such as norms, preparedness to take risk, and actual judgment of risk, which determine to some extent the listeners’ attitudes to loud music [160].

### 5.2. Music Consumption as Addictive Behavior

There is a possible analogy, in this regard, between music consumption and *addictive behavior* (see below), even if it does not clearly meet all criteria for addiction. Addiction has traditionally been defined as the pathological usurpation of neural processes that normally serve reward-related learning. Therefore, it can be considered as a maladaptive habit formation that involves the dopaminergic circuits of the brain, such as the nucleus accumbens, the ventral tegmental area, the dorsal striatum, and the prefrontal cortex [161]. Being related primarily to the mechanism of sensation seeking, with its correlates of animal excitement, it entails the danger of overstimulation and high arousal [157,158]. Such overstimulation can be situated in a culture of music or sound as power, echoing Phil Spector’s metaphor of a “wall of sound” [162], with a predilection for penetrating “hot” sounds and evoking the appearance of responses to addictive drugs, as mediated by vestibular–pharmacological activations. Moreover, the link between vestibular reward—through the activation of the saccule in the labyrinth of the inner ear—and addictive drugs has been observed in cases of loud sound environments, such as rock concerts and dance clubs, with sound levels in excess of 120 dB SPL [160], and the use of “dance drugs”, such as ecstasy and amphetamine, which increase the amount of available dopamine. Such loudness is experienced by some listeners as an intrinsic source of pleasure, as exemplified in what is commonly known as the “rock and roll threshold” of around 96 dB Leq [33,163]. It is suggested, therefore, that both acoustically evoked saccular responses and vibrotactile sensations could be considered as possible sources for the enjoyment of loud music, with an underlying hypothesis that acoustic saccular stimulation can be a substitute for the sensations of self-motion that can be obtained from swings, rocking chairs, and fun parks [164].The phenomenon is also related to motor entrainment, which plays a role in beat [165] and meter induction [166,167], and allows a rapid reward-based selection of the self-motion of the body in the sensory-motor circuits of the supplementary motor area (SMA) and the cingulate motor area (CMA) of the brain [165]. It makes the ability to align motor actions with external rhythms possible, which has proven throughout history to have the potential to hold groups together through coordinated rhythmic movement and the feelings it evokes.

Though somewhat elusive as a hypothesis, these claims have received some empirical evidence from neuroscientific research that found that triggering the saccule by using bass sounds—in particular low frequency and infrasound vibrations—results in bodily responses [168].

Conceiving of music as a “good drug”, however, is only as effective as listeners allow it to be, and, moreover, it may also result in negative side-effects [169]. Addiction—either in the positive or negative sense (see [170] who also coined the term “runner’s high”)—must always be defined in terms of costs and benefits. It has been questioned, in this regard, why listeners would enjoy stimuli that cause discomfort and negative impacts on their health [80,81]. There are, in fact, effects of noise exposure that include reversible and irreversible components, such as hearing threshold shifts, that can be temporary but can also progress for some years after exposure [154]. It is interesting, therefore, to approach the study of health-risk behaviors from the point of view of the *Social Ecological Model* [171,172], which is a framework that accounts for individual attitudes and beliefs and which also considers the impacts of the social environment. It states that individuals base their decisions about health on four levels of influence: the intrapersonal level of the person’s own thoughts and attitudes; the interpersonal level of influence from other people; the community level of cultural influences; and the policy level, with aspects of government policy and laws. The model has also been applied to the study of music listening by Welch and Fremaux [80,81]. In what they called the CAALM model—Conditioning, Adaptation and Acculturation to Loud Music—they investigated the reasons people enjoy loud sounds and music and found four main emerging themes: loud sound is arousing; it enables greater socialization; it masks unpleasant things; and it emphasizes personal identity.

These motivations are quite powerful, even so as to overrule the negative consequences of maladaptive listening strategies. This is the case, in particular, for listeners who rely on music to feel better and for those who use music in the pursuit of pleasure for its own sake. This last feature, which is also typical for addictive behavior, can lead to maladaptive patterns in the sense that addicts may come to “want” something without really “liking” it. This is not to be generalized to behavior overall, as it can be necessary to want something without liking it, such as the case of taking medicine for an ongoing medical condition. Yet, it reveals a possible dissociation between mechanisms that are largely subcortical (wanting) and those that are cortically-mediated, such as in conscious expectation and planning, which can be a direct route to great unhappiness [66].

### 5.3. From Hedonic Pleasure to Eudaimonic Enjoyment: Mindful Listening

This brings us to the role of *aesthetic emotions*, which may be hypothesized as having a place in the context of positive addiction but without the dangers of maladaptive listening. The claim, however, is not yet conclusive [26,173,174], though there is a lot of ongoing research that has been conducted in the context of so-called “peak experiences”. They are exemplified most typically in Konečni’s theory of the *aesthetic trinity*, which embraces the experience of awe, being moved, and thrills [105].

This tripartition provides an interesting starting point. There are, however, still ongoing debates about the construct validity of the terms, with the question of whether we should conceive peak pleasure as a unified psychological construct or as a set of distinct responses, which may vary in terms of elicitors, experiences, and individual differences between subjects, as a major distinction. The “chills”—as a subcategory of thrills—in particular, have been considered as the markers of emotional responses [145]. They can be measured in an objective way and can be differentiated by positive goose tingles and negative cold shivers, somewhat related to the distinction between approach and avoidance behavior. Moreover, chills experiences are multifaceted and may involve distinct feelings of awe, surprise, tension, pleasure, being moved, elevation, and nostalgia, which can be characterized either as positive and desirable or negative and aversive [175]. The concept of “being moved”—either joyfully or sadly—is otherwise based on the distancing–embracing model, which claims that negative states can be transformed into pleasurable experiences, such as aesthetic perceptions. Therefore, it should be considered as a mixed emotion, albeit predominantly positive [176,177].

It is interesting, in this regard, to distinguish between mere *hedonic pleasure* and *eudaimonic enjoyment*. Pleasure comes from the satisfaction of mere homeostatic needs, such as hunger, sex, and bodily comfort; enjoyment breaks through these constraints by stretching good feelings beyond immediate satisfaction, as exemplified in an outstanding athletic event, artistic performance, prosocial behavior, or stimulating conversation [65]. Both kinds of experience, however, are not opposed to each other. They may be complementary, as exemplified in the current definitions of well-being, which include both hedonic and eudaimonic aspects. It involves a subjective evaluation of the quality of life in terms of affective measures, a cognitive estimation of life satisfaction, and the assessment of the extent to which we are doing well rather than merely feeling good [58,68].

The aesthetic experience, with its reliance on aesthetic emotions, is closely linked to the experience of enjoyment [178]. It revolves around the conception of positive and negative affect, which are recognized as having adaptive functions in the sense that they may contribute to the building of cognitive and emotional resources [66]. As such, it is not to be equated univocally with skilled listening, as found in professional musicians or music scholars who may be reluctant to suspend their critical stance and tendency to judge.

It is possible, in this regard, to engage with music in a mindful way, relying mainly on some of the main attitudes and skills of *mindfulness*, such as the absence of evaluation or reacting in a habitual way to stimuli (non-judging), the practice of perceiving newness and a sense of wonder in an environment (beginner’s mind), the willingness to try new experiences and suspending a critical stance (suspending judgment), and the mental practice of experiencing without fighting or striving to change one’s current state (acceptance and letting go) [179,180]. Mindfulness skills, in this view, involve several components that open up possibilities for an increased sensitivity to psychological, somatic, and environmental cues, which are crucial for the operation of healthy regulatory processes: to observe and attend to the changing field of thoughts, feelings and sensations; to experience emotions with acceptance and nonjudgment; and to contribute to the regulation of the attention to be maintained on the immediate experience with an increased recognition of the mental events in the present moment, with an attitude of curiosity, openness, and acceptance [179,181,182,183,184].This focus on the present, with an awareness of the current experience, may contribute to a feeling of alertness and vigilance to what is occurring in the here and now. Central in this approach is the listener’s ability to enter a different relationship with his/her subjectivity and to learn to stand back and “reperceive” or “decenter”. This means that subjective qualia should be seen in a dispassionate way, as phenomena that pass through our internal world without identification or attachment [181,185]. It is a way to interrupt automatic maladaptive habits or, stated differently, conscious attention to and acceptance of experience in the moment that may engender a wider and more adaptive range of coping skills, which make it possible to experience strong emotions with a greater awareness, greater sense of disengagement, and less reactivity. Therefore, they increase resilience rather than investing in avoiding or denying behavior [185].

There is some empirical support from affective neuroscience and neuroaesthetics for these views. Aesthetic judgment shares its neural correlates in the reward system with *moral decision-making*, thus pointing in the direction of a common ground for aesthetic and moral judgments [186,187]. Activity in the medial orbito-frontal cortex occurs regardless of whether the source of the experience was visual, musical or mathematical [188,189,190]. These findings were a major starting point for later neuroimaging studies that revealed activity in the reward circuit as a whole as being a key component of the aesthetic experience. The orbitofrontal cortex is also implicated in emotional responses to music; it integrates sensory input with reward value and is integral to incorporating emotional and somatosensory input into decision-making processes [97,191]. It is also highly interconnected with the temporal pole, the amygdala, and the anterior insula, as discussed earlier, which suggests a role for their involvement in musico-affective experiences [44].

The relation of aesthetic judgment with *ethical* and *moral decision-making* and judgments, which weaves together emotion, high reasoning, creativity, and social functioning in a cultural context [6,192], is one of the most promising findings of current research. It gives new impetus to the old adage that music soothes the soul. Much is to be expected here from neuroimaging studies that focus on the effects of psychedelics, flow and meditation, and the experience of awe, which have been shown to be accompanied by a reduced activation of the default mode network [193]. It points into the domain of connectomics, which studies the brain networks and their mutual connections and neuroplasticity as related to the prolonged aesthetic experience of music [26,67]. Morphometric and histological studies, which portray the modification of white matter tracts, in particular, will prove to be of extreme importance in this regard [194,195].

## 6. Conclusions

In this paper we have argued for a biological and adaptive perspective on music listening. Starting from the claim that music listening is an evolutionary achievement that is based on cognitive and affective-emotional functions, we have tried to align it with the principles of homeostatic regulation. Adaptive listening, then, is a mechanism for coping with the sounds, which are not merely valued as an end in itself but also as a means for self-regulation and self-realization [196]. There is, therefore, a close relationship between music consumption and the reward system, with a possible transition from mere hedonic pleasure to eudaimonic enjoyment and a subtle interplay between arousal and valence as the major dimensions of core affect.

The role of the so-called aesthetic emotions is of primary importance here, in the sense that they may reflect adaptive ways of listening that turn out to have beneficial effects on our homeostatic functioning. Music, in fact, can also be seen as a potential stressor, which can be approached or avoided. It is tempting, in this regard, to rely on the neural correlates of music processing and to consider three levels of processing: the lower level processing in the brainstem; the affective-emotional processing in subcortical areas; and the higher-level elaboration in cortical areas. The final aim is to find linear-causal relations between acoustic stimuli and physiological and psychological reactions. Though this holds partially for lower-level reactivity to the sounds, there are so many modulating factors that intervene in the transition from sound to meaning, which makes this search for causal relations elusive. Yet, there is the challenging new emerging field of neuroaesthetics, which tries to generalize beyond the particular reactions of individual listeners in an attempt to provide operational descriptions of the so-called aesthetic experience of aesthetic stimuli in general, as well as in music. The study of the peak experiences of musical pleasure (chills, thrills, being moved) and the related study of the neurochemistry of musical emotions—with a special focus on the dopaminergic activity—have provided valuable insights into the effects of music on our body and mind. Moreover, the twin factors of valence and arousal have allowed a more objective study of the so-called aesthetic reactions, but they are also valuable in the study of the so-called maladaptive ways of listening. It seems that arousal is related primarily to the lower functions of the brain, while valence is mediated and modulated more profoundly by high cognition. The emotional-affective processing can be located between these levels, with many interconnections between the cortical and subcortical structures. A major finding in this regard is that neural activity in the reward circuit is a key component of the aesthetic experience, which means that music may activate neural circuits involved in emotion and reward [18].

These interconnections, however, are not to be taken for granted. They are the outcome of prolonged musical-aesthetic experiences which are able to alter both the structure and the functions of the brain [26,67]. There are, therefore, plastic changes which involve not only the grey matter of the brain but also the white matter, which means that the fibers and fasciculi that connect distinct areas of the brain are enlarged so as to assure stronger connections [194,195,197]; as evidenced by tractographic studies. As well as these morphometric findings about structural changes, there has been additional support from functional studies in the domain of connectomics [198], which studies the functional relations between spatially distributed areas of the brain. In this regard, the finding that there is a strong functional connectivity between the brain networks related to aesthetic judgment, evaluative and moral decision making, and the reward system is of major importance [66,88,173,186,187]. It is an insight that is growing in momentum and that fits perfectly with the above-mentioned transition from mere hedonic pleasure to eudaimonic enjoyment.

## Data Availability

Not applicable.
